# Investigation of strategies for the introduction and transportation of replacement gilts on southern Ontario sow farms

**DOI:** 10.1186/1746-6148-8-217

**Published:** 2012-11-09

**Authors:** Kate Bottoms, Zvonimir Poljak, Cate Dewey, Rob Deardon, Derald Holtkamp, Robert Friendship

**Affiliations:** 1 Department of Population Medicine, Ontario Veterinary College, University of Guelph, Ontario, Canada; 2 Department of Mathematics and Statistics, University of Guelph, Ontario, Canada; 3 Department of Veterinary Diagnostic and Production Animal Medicine, College of Veterinary Medicine, Iowa State University, Iowa, USA

**Keywords:** Swine, Biosecurity, Gilt replacement strategies, Multiple correspondence analysis

## Abstract

**Background:**

Porcine reproductive and respiratory syndrome (PRRS) is of major concern to the swine industry; infection with the virus can lead to production losses, morbidity, and mortality within swine operations. Biosecurity practices related to the management of replacement animals are important for the prevention and control of the PRRS virus, as well as other diseases. The objectives of this study were: (i) to describe individual biosecurity practices related to the introduction and transportation of replacement gilts on southern Ontario sow farms, and (ii) to understand patterns in the implementation of these practices. The second objective was accomplished using multiple correspondence analysis (MCA), which allows visualization of the relationships between individual practices and provides information about which practices frequently occur together, and which practices rarely occur together. These patterns constitute strategies for the implementation of biosecurity practices related to the introduction and transportation of replacement gilts. Data were collected using version 2 of the Production Animal Disease Risk Assessment Program’s survey for the breeding herd. Two subsets of variables were retained for analysis; one subset pertained to how replacements were managed upon arrival to the farm, and the other pertained to the transportation of genetic animals.

**Results:**

For both subsets of variables, the results of the MCA procedure were similar; in both solutions the 1st dimension separated herds that were closed with respect to replacement animals from herds that were open, and the 2nd dimension described how open herds managed replacements. The most interesting finding of this study was that, in some cases where a risky practice was being implemented, it was closely associated with other biosecurity practices that may mitigate that risk.

**Conclusions:**

The findings from this approach suggest that one cannot always examine biosecurity on a variable-by-variable basis. Even if a practice that is generally considered high-risk is being implemented, it may be balanced by other practices that mitigate that risk. Thus, the overall biosecurity strategy on a farm must be considered instead of only examining the implementation of individual practices.

## Background

Porcine reproductive and respiratory syndrome (PRRS) virus is of major concern to the swine industry worldwide. Clinical signs associated with infection include inappetance, lethargy, depression, pyrexia, respiratory distress, premature farrowing, and increases in stillborn or poor-doing piglets and in pre-weaning mortality [1]. Direct contact between an infected, pathogen-shedding pig and one that is susceptible to infection is the most important route of transmission for most infectious diseases of swine, including the PRRS virus (PRRSV) [2-4]. Given the importance of this virus to the swine industry and its potential impact on the welfare and productivity of pigs, gilt replacement strategies are essential for the prevention and control of PRRS [5]. Such strategies pertain to the source from which replacements are obtained, how replacement gilts are managed upon arrival to the farm, and the protocols used for their transportation. Isolation and acclimation facilities are particularly important because they allow for separation between replacement animals of unknown disease status and the main herd. One recent study examined gilt replacement strategies in Quebec and provided descriptive information about practices related to the purchase and introduction of gilts [5]. The authors found that some practices were not adequately applied, and identified specific factors that may increase the risk of PRRSV introduction or re-circulation in sow herds [5].

Previous work by this research group found that external biosecurity on sow farms in southern Ontario was best described by three groups: two of which were considered to have high biosecurity standards, and one of which was considered to have low biosecurity standards. The most important variables in differentiating between these groups related to the source of replacement animals and transportation practices [6]. Thus, the objectives of this study were: (i) to describe individual biosecurity practices related to the introduction and transportation of replacement gilts on southern Ontario sow farms, and (ii) to understand patterns in the implementation of these practices.

## Methods

### Questionnaire

Information about breeding animal replacement strategies on sow farms in southern Ontario was obtained from the Production Animal Disease Risk Assessment Program’s (PADRAP) survey for the breeding herd [7]. This survey was originally developed to assess the risk of PRRSV introduction and spread within a herd and is widely used by swine practitioners in North America. Version 2 of the survey was used for the study; this version contains 179 questions and is divided into three sections that address demographic information, internal risks, and external risks.

### Herd inclusion and interviews

The source population for this study was southern Ontario sow herds. The sampling strategy for data collection has been reported previously [6]. In brief, herds were eligible for inclusion in the study if they were located in southern Ontario and if sows were present on-site. Information about the study was communicated at the Ontario Association of Swine Veterinarians (OASV) meetings and through the listserv. Swine veterinarians provided contact information for herds that were eligible to participate. A total of 161 swine sites were included in the study, and the study period ran from April through August of 2007. Interviews were conducted by three veterinary students who had previous experience in the swine industry. When required, additional information regarding disease status was obtained from the herd veterinarian.

### Multiple correspondence analysis

Multiple correspondence analysis (MCA) (SPSS 19.0, SPSS Inc., Chicago, IL) is a multivariate technique that is used to visualize relationships within a set of categorical variables [8]. This method is often used to analyze survey data, where each variable corresponds to a question, and the categories of each variable correspond to the possible responses [9]. There is no outcome variable with this particular method; instead, the procedure identifies patterns among responses to the selected variables. Variables that are used in the MCA solution, such as biosecurity practices, are classified as “active” variables. This method also allows the use of “supplementary” variables; these variables are not used in the calculations and have no influence on the solution, but are displayed on the output and aid in interpretation of the active variable categories [8,10].

In the MCA output, relationships between different categories of the selected variables are typically represented as points in a two-dimensional space; the first and second dimensions being the X and Y axes of the graph. As it pertains to this study, biosecurity practices that tend to occur together are plotted close to each other, and biosecurity practices that rarely occur together are plotted further apart [10]. The output plot is usually organized such that each variable is assigned a symbol, and each of the responses to that particular variable are labelled beside each of the symbols. For example, if a variable of interest is assigned a black square and has 3 possible responses, the plot will contain 3 black squares, each labelled to indicate which variable category it refers to. The plot is interpreted by considering which variable categories are plotted closely together; relatedness between variables is considered in both the 1st dimension along the X axis, and in the 2nd dimension along the Y axis. Similarly, the distribution of observations that are similar is plotted as part of the MCA output; this provides information about which herds are similar in the 1st the 2nd dimensions.

This method has been used previously to describe biosecurity and management practices in Belgian swine herds [10]. In the current study, variables relating to both internal and external risk were selected for MCA. Variables of interest were subdivided into two groups: those pertaining to how replacements were handled upon arrival to the recipient farm, and those pertaining to the transportation of replacements. Introduction variables provided information about the number of replacement sources in the previous 2 years; where replacements were obtained from; PRRSV status of the source herds(s); whether replacements were exposed to the herd strain of PRRSV via natural or controlled exposure; and how replacements were handled in terms of isolation and acclimation (Table 1). Transportation variables addressed the frequency of replacement deliveries to the site; the cleaning and disinfection of trucks carrying genetic animals; and whether there were flow, route, transit, or use restrictions during transportation (Table 2).

**Table 1:** Active variables used in multiple correspondence analysis for the introduction of replacement animals, on 161 southern Ontario sow farms

**Variable**	**Categories**	**Value label**	**Percentage**
a. Time (days) between last natural exposure of replacements to live animals or feedback and entry into breeding herd	0 days	5	20.5%
	1 to 60 days	4	6.8%
	61 to 90 days	3	1.9%
	91 days or more	2	0.6%
	Not applicable	9	70.2%
b. Replacements are exposed to serum from viremic pigs or sows via injection prior to entry	Yes	5	14.3%
	No	1	85.7%
c. Time (days) between last exposure to injected serum and entry of replacements into breeding herd	0 days	5	1.9%
	1 to 60 days	4	5%
	61 to 90 days	3	4.3%
	91 days or more	2	0.6%
	Not applicable	9	88.2%
d. Number of breeding herd sources from which replacements have been obtained in last two years	4 or more	5	1.9%
	3	4	0.6%
	2	3	13%
	1	2	64%
	0	1	20.5%
e. Source of replacement animals	Some or all purchased from other production systems/genetic suppliers	5	52.2%
	Some or all from other sites outside the pig flow but within the same production system, none from outside the production system	4	4.3%
	Some or all from other sites within the same pig flow as this site (e.g., downstream nursery or grow/finish/developer), none from outside the same pig flow	3	3.7%
	Closed herd at this site (replacements are born at site, moved to another site and later returned as replacements)	2	9.3%
	Closed site (replacements are born and raised at site and never moved from site)	1	30.4%
f. PRRS virus status of breeding herd(s) from which replacements are sourced	One or more sources positive active – this is positive by ELISA and producing PRRSV infected weaned pigs	5	8.7%
	One or more sources with unknown status, none positive active	4	1.9%
	One or more sources positive stable – that is positive by ELISA but producing non-infected weaned pigs, none positive active or unknown status	3	34.2%
	All sources currently negative by ELISA but one or more have been positive in the past	2	40.4%
	All sources have always been negative (naive)	1	14.9%
g. PRRS virus status of breeding female replacements in isolation/acclimation	Negative at entry but field virus positive from natural exposure at exit	5	18.6%
	Field virus positive from natural exposure at entry	4	15.5%
	Negative at entry & negative at exit	3	29.2%
	Not applicable	9	36.6%
h. Response when group of replacement animals in isolation/acclimation becomes positive by PCR or ELISA to PRRS virus from natural field virus exposure	Introduced into breeding herd on regular schedule	5	14.9%
	Introduced into breeding herd after holding period of less than 30 days	4	4.3%
	Introduced into breeding herd after 30 to 90 day holding period	3	11.2%
	Introduced into breeding herd after holding period of more than 90 days	2	2.5%
	Replacements are marketed and not used for breeding purposes	1	26.1%
	Not applicable	9	41%
i. Isolation/acclimation period (days)	0 or less	5	41.3%
	1 to 60 days	4	41.3%
	61 to 90 days	3	8.8%
	91 to 120 days	2	5.6%
	121 days or more	1	3.1%
j. Replacement animal acclimation flow	Continuous flow	5	28%
	All in/all out by room	4	3.7%
	All in/all out by barn	3	3.1%
	All in/all out by site	2	0.6%
	Not applicable	9	64.6%
k. Replacement animal isolation flow	Continuous flow	5	20.5%
	All in/all out by room	4	12.4%
	All in/all out by barn	3	13.7%
	All in/all out by site	2	0.6%
	Not applicable	9	52.8%
l. Location of replacement animal acclimation housing relative to this site	On-site in same air space as sow herd	5	19.3%
	On-site in different air space as sow herd	4	10.6%
	Off-site (different site from sow herd)	3	5.6%
	Not applicable	9	64.6%
m. Location of replacement animal isolation housing relative to this site	On-site in same air space as sow herd	5	4.3%
	On-site in different air space as sow herd	4	28.6%
	Off-site (different site from sow herd)	3	14.9%
	Not applicable	9	52.2%
n. Serum testing of replacement animals for PRRS virus or antibodies by PCR or ELISA upon entry into acclimation/isolation site(s)	No routine testing done	5	66.5%
	A sample subset of incoming animals are tested upon entry	4	4.3%
	All incoming animals are bled and tested upon entry	3	0.6%
	Not applicable	9	28.6%
o. Serum testing of replacement animals for PRRS virus or antibodies by PCR or ELISA upon exit from acclimation/isolation site(s)	No routine testing done	5	59%
	A sample subset of incoming animals are tested upon entry	4	11.8%
	All incoming animals are bled and tested upon entry	3	1.9%
	Not applicable	9	27.3%

Note: for some variables, there may have been additional response options that were not selected by any of the herds in our sample. These categories are not presented in the table.

**Table 2:** Active variables used in multiple correspondence analysis for the transportation of replacement animals, on 161 southern Ontario sow farms

**Variable**	**Categories**	**Value label**	**Percentage**
p. Frequency of replacement deliveries to this site (days between deliveries)	30 or less	5	37.3%
	31 to 45 days	4	6.2%
	46 to 60 days	3	18%
	61 to 90 days	2	10.6%
	91 days or more	1	7.5%
	Not applicable	9	20.5%
q. Flow restrictions on vehicles used to transport genetic animals	No restrictions, the same vehicle may haul PRRSV positive and negative animals	5	14.3%
	The same vehicle can haul PRRSV positive and negative animals but a minimum downtime is required before visits to negative sites following last visit to positive site	4	19.9%
	The same vehicle never hauls both PRRSV positive and negative animals	3	21.7%
	Truck(s) are dedicated to this site and do not haul animals from other sites	2	44.1%
r. Route restrictions on vehicles used to transport genetic animals	No special route selection practices	5	70.2%
	Transport routes are outlined proactively to avoid roads with swine and swine-related sites along the route	1	29.8%
s. Transit restriction on vehicles used to transport genetic animals	Transport vehicles are allowed to stop en route	5	42.2%
	Transport vehicles are allowed to stop en route only at designated times and locations	4	8.1%
	Transport vehicles are never allowed to stop en route	3	49.7%
t. Use restrictions on vehicles used to transport genetic animals	Vehicles used to transport genetic animals to and from other sites within the production system may transport non-genetic animals or animals to market or collection points	5	32.9%
	Vehicles used to transport genetic animals to and from other sites within the production system are not used to transport non-genetic animals or animals to market or collection points	1	67.1%
u. Washing frequency of vehicles used to transport genetic animals	Never, rarely, or unknown	5	1.9%
	At least once per 20 loads	4	2.5%
	At least once per 10 loads	3	8.8%
	Between every load	2	48.1%
	Not applicable	9	38.8%
v. Pre-rinse with water to flush away loose organic material prior to wash of vehicles used to transport genetic animals	Unknown	5	8.8%
	No, pre-rinse not done	4	3.8%
	Yes, fresh water used	3	48.8%
	Not applicable	9	38.8%
w. Disinfectant use on vehicles used to transport genetic animals	No disinfectant used or unknown	5	25%
	Phenol-based compound (BioPhene, Environ, Tek-Trol, Laro, Lysol) or aldehydes (DC&R, Cidex, Formaldegen) used	4	3.1%
	Quarternary ammonium (Roccal, Germex, Zephiran, Hi-Lethol, BioSentry) used	3	2.5%
	Hypochlorite (Clorox, Halazone, Chloramine-T) or peroxygen (Virkon) used	2	5.6%
	Iodine (Wescodyne, Premise, Iofec, Iosdyn, Losan) or quarternary ammonium combinations (Synergize, Aseptol) used	1	25%
	Not applicable	9	38.8%
x. Drying time following wash of vehicles used to transport genetic animals	No requirements	5	3.8%
	Vehicles allowed to dry completely before next load	4	53.8%
	Assisted drying technology is used to dry washed vehicles	3	5.6%
	Not applicable	9	36.9%

Note: for some variables, there may have been additional response options that were not selected by any of the herds in our sample. These categories are not presented in the table.

The PADRAP questionnaire was designed such that responses are ordered from highest to lowest risk; we maintained the original ordering, and a value of 5 was assigned to the most risky behaviour. The remaining categories were assigned values in descending order. In the case of dichotomous variables, a value of 5 was assigned to the response with the highest risk, and a value of 1 was assigned to the response with the lowest risk. Reponses of “not applicable” were assigned a value of 9, in order to make our interpretation of the MCA plot straightforward.

In both MCA solutions, two supplementary variables were included (Table 3): PRRSV status and biosecurity group membership. Each farm was defined as PRRSV-positive, negative or naïve at the time the interview was conducted. According to this version of the PADRAP questionnaire, a positive status indicates that the herd was positive on ELISA and may or may not have been producing infected weaned pigs. A negative status indicates that the herd still contained previously exposed animals, and a naïve status indicates that the entire herd had never been exposed to the PRRS virus. Information regarding biosecurity group membership for each of the farms was obtained from previous work by this research group. In that study, the same dataset set was used, and a different subset of variables was offered to cluster analysis. Three external biosecurity groups were identified and named by the authors as: (i) high biosecurity herds that were open with respect to replacement animals; (ii) high biosecurity herds that were closed with respect to replacement animals; (iii) low biosecurity herds [6].

**Table 3:** Supplementary variables used in both multiple correspondence analysis solutions, on 161 southern Ontario sow farms

**Variable**	**Categories**	**Percentage**
PRRS status	Positive	66.5%
	Negative	21.7%
	Naïve	11.8%
Biosecurity group	High biosecurity (open)	39.8%
	High biosecurity (closed)	26.1%
	Low biosecurity	34.2%

## Results

### Demographic information

Our sample of 161 sow herds consisted of 3 herd types: 45.3% were farrow-to-finish, 42.9% were farrow-to-wean, and 11.8% were farrow-to-feeder operations. The number of sows on the premises ranged from 45 to 3500, with a mean of 800.3. In terms of production type, 81.1% of the farms included in the study were commercial operations, and 18.9% were breeding herds that produced animals for replacement and genetic improvement. Information about the supplementary variables is presented in Table 3. Of the herds that were positive for the PRRS virus, 81.3% were stable, 15.9% were unstable, and 2.8% were of unknown stability.

### Multiple correspondence analysis for the introduction of replacement gilts

**Figure 1:**
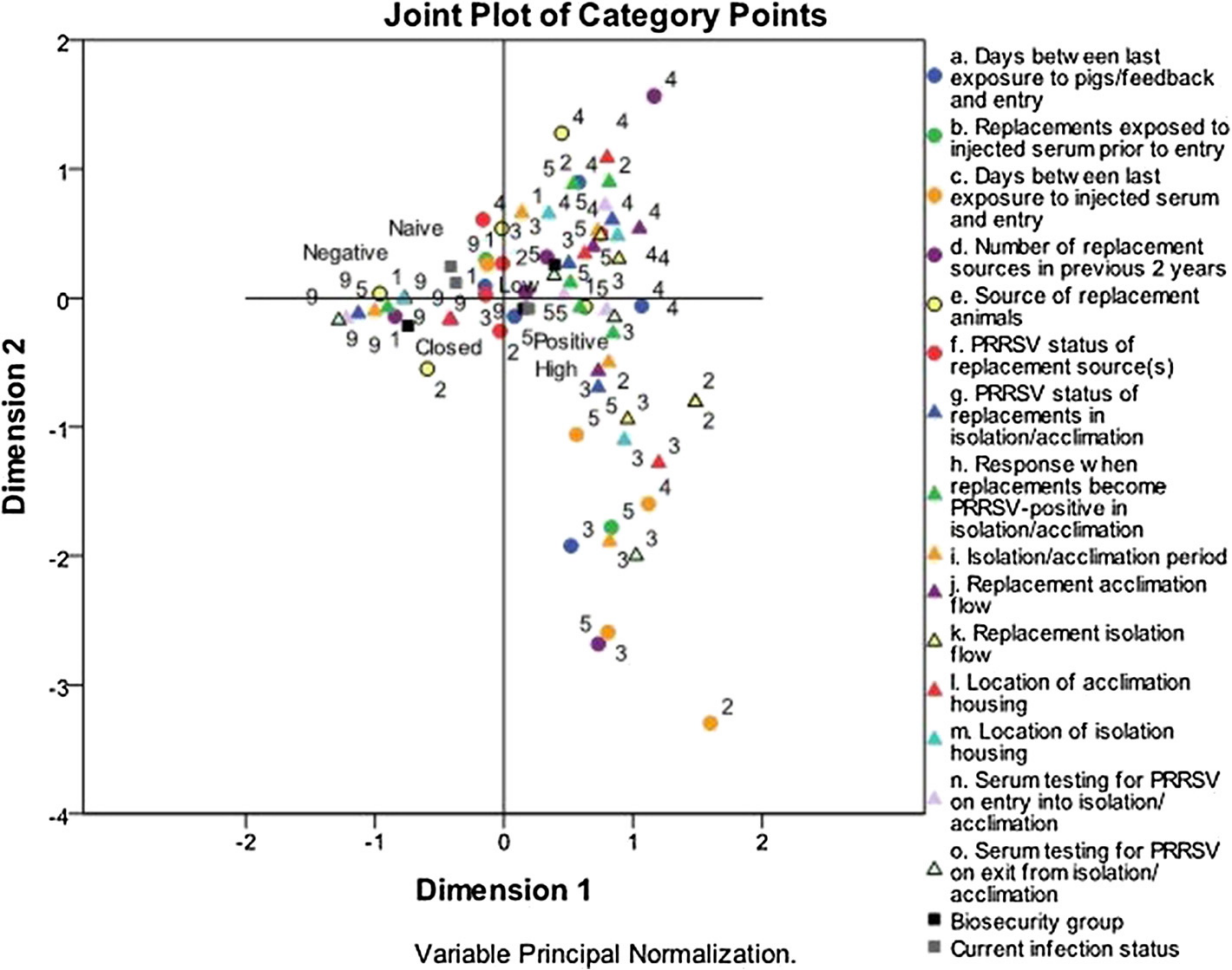
**Multiple correspondence analysis solution for the management of replacement animals upon arrival to the farm, on 161 southern Ontario sow farms.** Different categories of biosecurity practices are displayed in this two-dimensional solution that explains most of the variability in the data. Categories occur closely together if they are correlated in their respective dimensions. The categories of each variable are labelled in descending numerical order, with a value of 5 indicating the practice considered to have the highest risk. See Table 1 for a description of which value label corresponds to which category.

**Figure 2:**
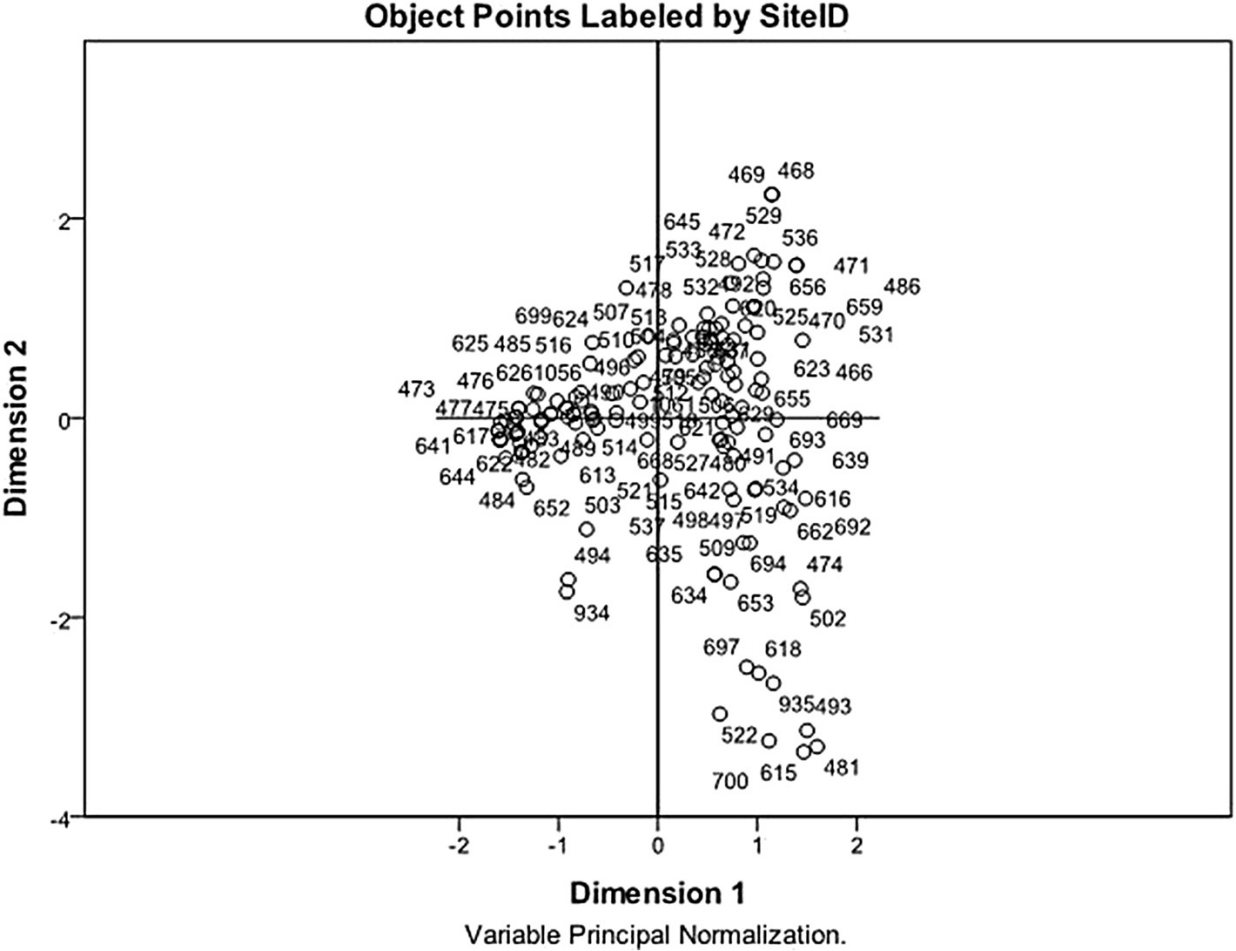
**Distribution of the 161 sow herds used in the multiple correspondence analysis solution for the management of replacement animals upon arrival to the farm.** The numbers on the plot correspond to the herd identification number, and herds that occur closely together have similar strategies for the introduction of replacement animals.

Variables pertaining to the introduction of replacement gilts are presented in Table 1. The MCA solution is presented in Figure 1, and the plot showing the distribution of herds is presented in Figure 2. The total variance explained by the solution was 63.7%, with 42.7% explained by the 1st dimension and 21% by the 2nd dimension. Discrimination measures provide insight into the influence exerted by each variable [10]. Variables with the highest discrimination are presented in Table 4.

**Table 4:** Discrimination measures of variables that were used in multiple correspondence analysis of the introduction and transportation of replacement animals, on 161 southern Ontario sow farms, the variables with the 5 highest discrimination measures are presented

**Solution**	**Variable**	**Discrimination measure**	
		**Dimension 1**	**Dimension 2**
Introduction variables	PRRS virus status of breeding female replacements in isolation/acclimation	0.749	0.173
	Isolation/acclimation period (days)	0.724	0.452
	Location of replacement animal isolation housing relative to this site	0.673	0.266
	Replacement animal isolation flow	0.664	0.185
	Serum testing of replacement animals for PRRS virus or antibodies by PCR or ELISA upon exit from acclimation/isolation site(s)	0.643	0.103
	Time (days) between last exposure to injected serum and entry of replacements into breeding herd	0.127	0.569
	Replacements are exposed to serum from viremic pigs or sows via injection prior to entry	0.116	0.527
	Location of replacement animal acclimation housing relative to this site	0.338	0.258
Transportation variables	Pre-rinse with water to flush away loose organic material prior to wash of vehicles used to transport genetic animals	0.957	0.346
	Washing frequency of vehicles used to transport genetic animals	0.956	0.285
	Disinfectant use on vehicles used to transport genetic animals	0.956	0.558
	Drying time following wash of vehicles used to transport genetic animals	0.921	0.171
	Flow restrictions on vehicles used to transport genetic animals	0.809	0.330
	Transit restriction on vehicles used to transport genetic animals	0.253	0.426
	Route restrictions on vehicles used to transport genetic animals	0.043	0.287

The 1st dimension separates practices related to herds that are closed with respect to replacement animals from practices related to herds that are open. The most informative variable in this respect is the source of replacement animals. On the left side of the 1st dimension are variable categories associated with closed herds: “closed site (replacements are born and raised at site and never moved from site)” and “closed herd at this site (replacements are born at site, moved to another site and later returned as replacements)”. On the right side of the 1st dimension are categories associated with open herds: “some or all from other sites outside the pig flow but within the same production system, none from outside the production system” and “some or all purchased from other production systems/genetic suppliers”. The intermediate category of “some or all from other sites within the same pig flow as this site (e.g., downstream nursery or grow/finish/developer), none from outside the same pig flow” is located exactly in the centre. Other variables confirm this separation of open and closed herds along the 1st dimension. The left side of this plot is dominated by responses of “not applicable” for all variables pertaining to the isolation and acclimation of replacement animals, and contains the response of “zero replacement sources in the last 2 years”. Alternatively, the right side of the 1st dimension contains practices that are associated with open herds. For example: for the location of isolation housing, responses of “on-site” and “off-site” are located on the right side of the plot, whereas the response of “not applicable” is located on the left. The closed biosecurity group, and PRRSV-negative and naïve categories also fall in this region of the plot. The right side of the 1st dimension includes the high and low biosecurity groups, and the PRRSV-positive category.

The 2nd dimension provides insight into how open herds manage replacement animals. In this plot, the lower right quadrant generally represents the least risky response patterns. The number of replacement sources in the previous 2 years, at 4 or more, was high. However, the risk posed by the high number of replacement sources was mitigated by several factors. These herds did well in terms of having a long period of time between the last exposure of replacements to either live animals, feedback, or injected serum from viremic pigs, and entry to the main herd. The isolation and acclimation period was long, with the facilities located off-site. Additionally, all replacements were blood tested for the PRRS virus upon exit from the isolation/acclimation facilities. The use of such high standards in the management of replacement animals mitigates the risk associated with using multiple replacement sources. The PRRSV-positive category is located in this quadrant, in close proximity to the high biosecurity group. About 80% of the PRRSV-positive herds in our sample were stable.

Alternatively, the most risky responses for the introduction of replacements tended to fall in the upper right quadrant of Figure 1. In this region, several risky practices are grouped together: the use of 3 replacement sources in the previous 2 years, some or all of which were from other sites outside of the same pig flow, combined with isolation and acclimation facilities located on-site, is concerning. An isolation/acclimation period of zero days was found in this quadrant, indicating that in some cases replacements are introduced directly to the main herd. The low biosecurity group was located in this quadrant.

### Multiple correspondence analysis for the transportation of replacement gilts

**Figure 3:**
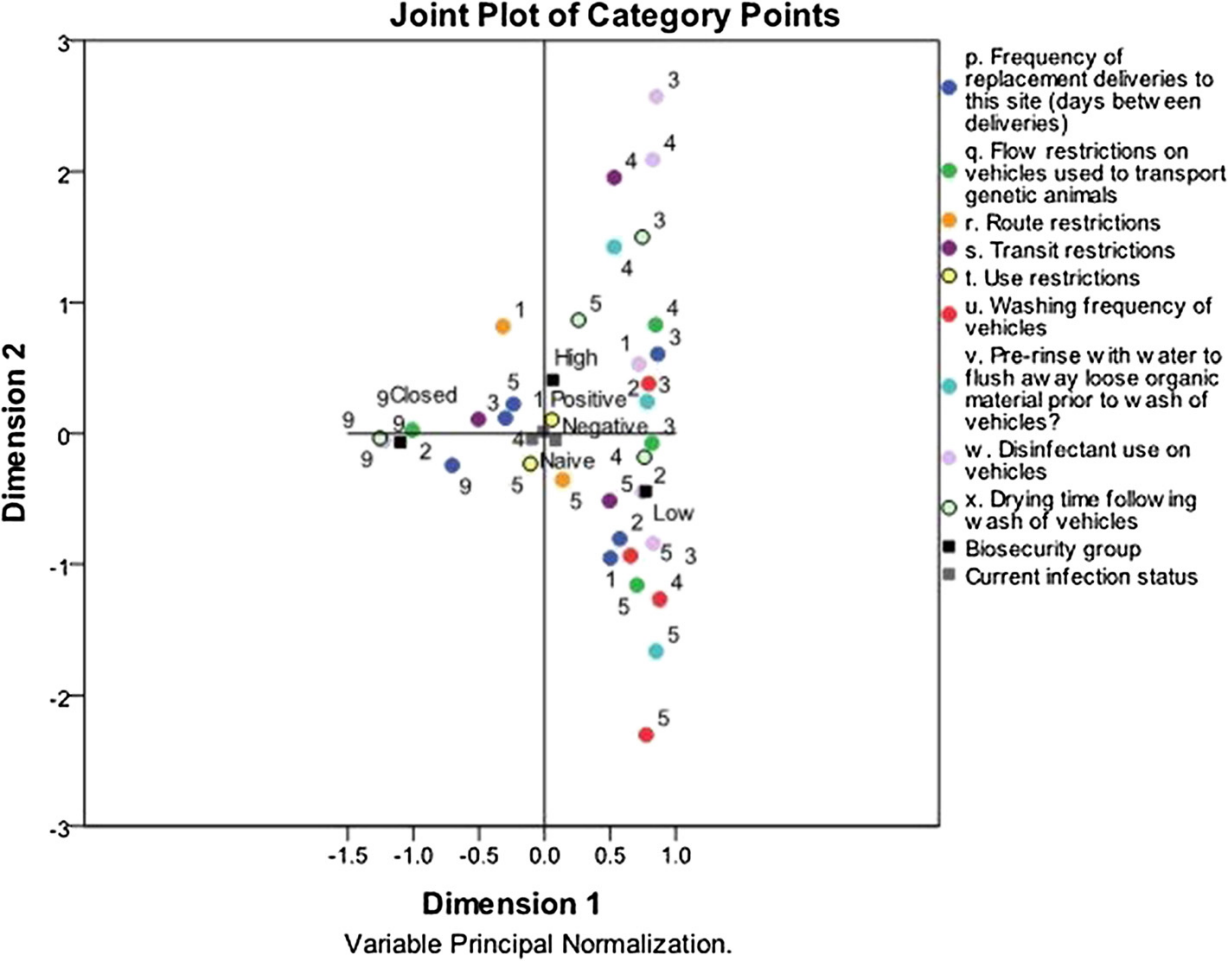
**Multiple correspondence analysis solution for the transportation of replacement animals, on 161 southern Ontario sow farms.** Different categories of biosecurity practices are displayed in this two-dimensional solution that explains most of the variability in the data. Categories occur closely together if they are correlated in their respective dimensions. The categories of each variable are labelled in descending numerical order, with a value of 5 indicating the practice considered to have the highest risk. See Table 2 for a description of which value label corresponds to which category.

**Figure 4:**
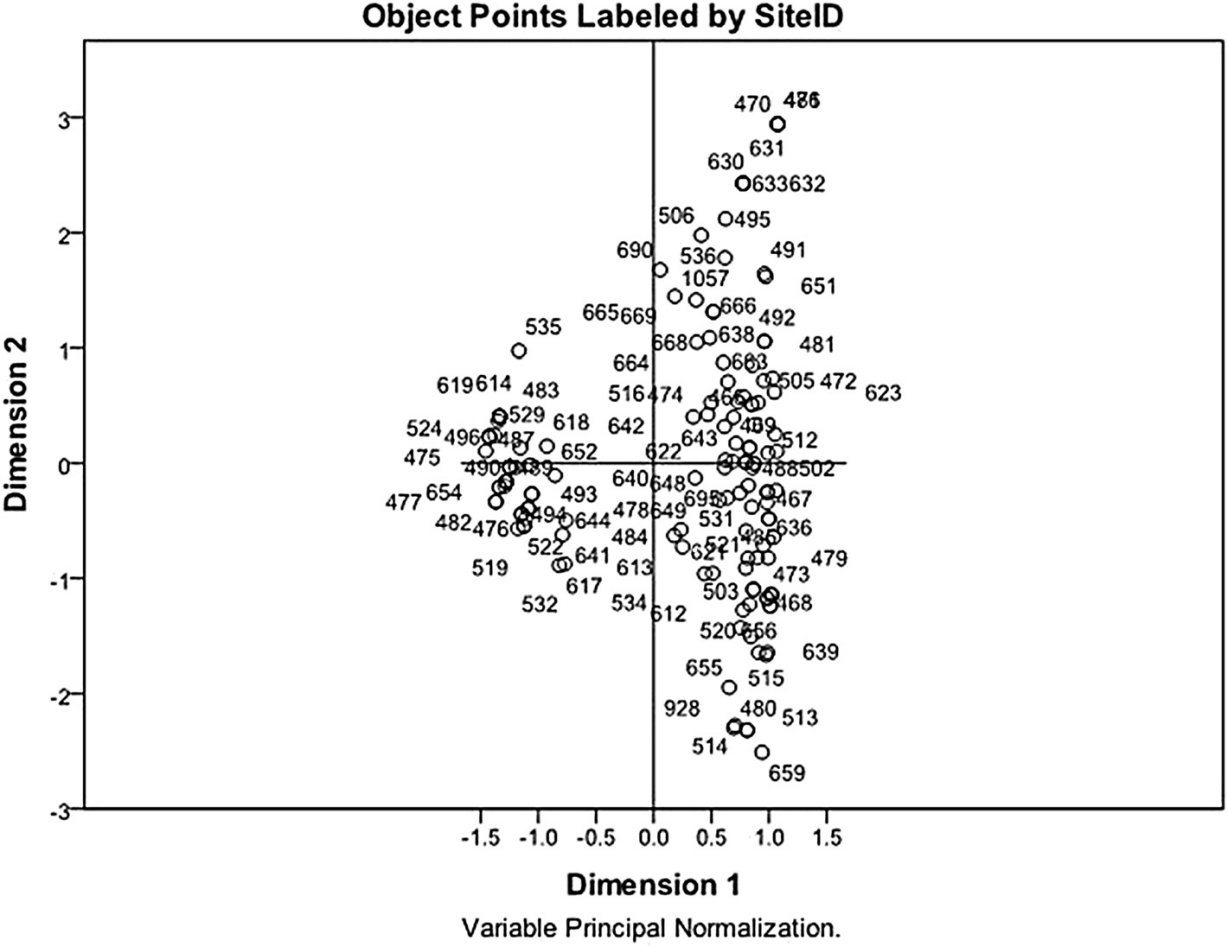
**Distribution of the 161 sow herds used in the multiple correspondence analysis solution for the transportation of replacement animals.** The numbers on the plot correspond to the herd identification number, and herds that occur closely together have similar strategies for the transportation of replacement animals.

Variables used to address strategies for the transportation of replacement gilts are presented in Table 2. The MCA solution is presented in Figure 3, and the plot showing the distribution of herds is presented in Figure 4. The total variance explained by the solution was 87.5%, with 58% explained by the 1st dimension and 29.6% by the 2nd dimension. Variables with the highest discrimination are presented in Table 4.

The 1st dimension, as in the previous solution, separates practices related to herds that are closed with respect to replacement animals from practices related to open herds. The left side of the 1st dimension contains responses associated with closed herds, while the right side of the first dimension contains practices that are associated with open herds. For example: for the variable regarding the washing frequency of vehicles used to transport genetic animals, responses of “never, rarely, or unknown”, “at least once per 20 loads”, “at least once per 10 loads”, and “between every load” are located on the right side of the plot, while the response of “not applicable” is located on the left. This pattern occurs for variables pertaining to the frequency of replacement deliveries, whether trucks are pre-rinsed to remove organic material, whether disinfectant is used, and regarding drying time following wash of vehicles. In regards to truck flow, the practices occurring on the left side of the plot indicate that trucks transporting genetic animals were dedicated to the site and did not haul animals from other sites. The closed biosecurity group falls in this region of the plot.

The 2nd dimension of this plot demonstrates how open herds manage the transportation of replacement animals; practices on either side of the axis represent relatively different strategies. The upper right quadrant of this plot represents the least risky responses. Although the same vehicle was allowed to transport both PRRSV-positive and negative animals, a minimum downtime was required following a visit to a positive site, vehicles were washed between every load, and trucks were disinfected using phenol-based compounds, aldehydes, quarternary ammonium, iodine, or quarternary ammonium combinations. One study evaluated the efficacy of disinfectants in PRRSV-contaminated transport vehicles, and found that the virus did not persist in trailers treated with Syngerize and Aseptol disinfectants, which are quarternary ammonium combinations [11]. The high biosecurity group was also located in this quadrant.

Alternatively, the most risky responses for all but two variables fall into the lower right quadrant. In this region of the plot, several inferior practices are grouped together: the combination of a lack of flow restrictions on trucks carrying replacement animals, with infrequent washing of trucks between loads and the lack of disinfectant use are particularly concerning. Also in this quadrant are the practices of 61–90 days and 91 days or more between deliveries of replacement animals. These are considered to be the least risky responses, but this finding is likely related to herd size. Indeed, when herd size and frequency of replacement deliveries are compared using the Kruskall-Wallis test, there is a significant difference in herd size between the different categories of the frequency of replacement deliveries (*P* < 0.05). When these same variables are cross-tabulated, the categories of 61–90 days and 91 days or more between replacement deliveries were associated with the smallest mean herd sizes.

## Discussion

In both MCA solutions the 1st dimension was primarily related to whether a herd was open or closed with respect to replacement animals, and the 2nd dimension described how replacement animals are handled in open herds. The absence of discrete patterns on these plots indicates broad variation in how different protocols are applied [10]. The 3rd dimension may provide more insight into these relationships, however we can obtain useful information based on the general differences between the extremes. In both MCA solutions, better practices are grouped in one quadrant, poorer practices are grouped in another quadrant, and practices that are associated with herds being closed with respect to replacement animals are grouped together on the left side of the plot. In previous work by this research group, two-step cluster analysis identified three external biosecurity groups using a different subset of variables from the same dataset [6]. Herds belonging to the high biosecurity group that was closed with respect to replacement animals generally did not receive replacements from outside the production system, and the movement of animals was usually via dedicated trucks. Herds belonging to the high biosecurity group that was open with respect to replacement animals generally had higher trucking standards for the movement of animals and feed, and higher entrance sanitation requirements. Herds belonging to the low biosecurity group tended to rely on replacement animals from outside the production system, and did not have strict policies regarding the trucking of live animals or feed [6]. In the current study, the locations of the biosecurity groups on both MCA plots agree well with previous work. The high biosecurity group that was closed with respect to replacement animals was closely associated with responses of “not applicable” for the introduction and transportation of replacement animals. In both MCA plots, the high biosecurity group that was open with respect to replacement animals was located in the quadrant with the least risky strategies for the handling of replacement animals, whereas the low biosecurity group was located in the quadrant with the most risky set of strategies.

The most important finding of this study is the understanding of how individual biosecurity practices form biosecurity strategies, particularly with respect to the introduction of replacement gilts into sow herds. Within these strategies, we can expect to find some practices that are generally considered high-risk, accompanied by other biosecurity practices that mitigate the risk. For example, in the MCA solution concerning the introduction of replacement gilts, the practice of having 4 or more replacement sources in the previous 2 years was closely associated with biosecurity practices that mitigated the associated risk, such as moderate isolation and acclimation periods that occurred in facilities located off-site, and blood testing of all replacements for PRRSV upon exit from the isolation/acclimation facilities. In order to ensure replacements are not viremic, it is advised that all replacement gilts are tested prior to entry into the main herd [5]. The use of multiple sources for replacement animals may be a necessity created by modern pork production systems, in order to facilitate improvements in the breeding program; the strategy described above mitigates the risk posed by this practice. At the other extreme are herds that were obtaining gilts from within the production system, some of which were introducing them after an isolation/acclimation period of between 1 and 60 days. Regardless of the source, isolation and acclimation of replacements is essential. Incoming pigs may appear healthy but be incubating infection or acting as carriers of a pathogen, and the likelihood of transmission to susceptible pigs is increased by the stress inherent to loading, mixing, and transportation of these animals [12,13]. Even when using PRRSV-negative suppliers, pigs may come into contact with the virus during transportation [5]. In the MCA solution concerning the transportation of replacement animals, some regions of the plot contain both good and poor practices in close proximity. Although the 3rd dimension may provide more insight into these relationships, generally these inconsistencies indicate broad variation in application of biosecurity protocols in this particular quadrant of the MCA plot. It is important to consider that vehicles that transport livestock are known to play a role in the spread of PRRSV from contaminated premises, and vehicle cleanliness is important in mitigating the associated risk [3,14].

The complex interrelationships between these biosecurity variables cannot be easily examined by correlation coefficients alone, and MCA has proved useful in that respect. The findings of this study could have important implications for the assessment of biosecurity practices, since our results suggest that infection control should not rely exclusively on the benchmarking of individual practices against an ideal standard. Additionally, the entire strategy should be assessed simultaneously; the implementation of such strategies is likely driven by their feasibility, cost, and effectiveness. The practical application of this finding is that standard-setting agencies should not only look at promoting specific individual biosecurity practices. Groups of practices that form strategies should be examined in order to determine whether the strategy is designed to effectively reduce the risk of introducing pathogens. This idea aligns with the principle of equivalence set forward by the World Organization for Animal Health, which recognizes that different approaches to animal health and production systems can provide equivalent animal and human protection for the purposes of international trade [15]. Thus, encouraging producers to apply the same biosecurity standard for every individual practice may be an over-simplified approach for some aspects of biosecurity, such as the introduction of replacement gilts.

The results obtained from this study are subject to some limitations. The herds used in this study represent a convenience sample that was recruited without a formal selection process; this may be the biggest limitation of the study. We are unable to provide response rates, as this study was an industry-based project, and those statistics were not available to us. Nonetheless, the variation in herd size within our sample indicates that a broad variety of management styles were included in the study. Additionally, participation was voluntary, and although not a specific requirement, herds were more likely to be selected for participation if their veterinarian was a member of OASV. Our study group may differ from the source population as a result of these potential selection biases [16]. As with any survey data, the use of closed-ended questions may mean that some information regarding gilt replacement strategies could have been misclassified, resulting in a potential bias [16]. Our decision to include the external biosecurity groups from previous work may also serve as a limitation of this study. Some of the variables used in the two-step cluster analysis were also used in the MCA solutions presented here. For the introduction strategies, 4 variables were used in both methods; for the transportation strategies, 2 variables were used in both methods. This was done to allow a more complete assessment of biosecurity, as it relates to these two important areas. Additionally, the biosecurity groups were not used in the MCA solutions; they were supplementary variables that aided in our interpretation of the plots.

## Conclusions

In both MCA solutions, there was a clear distinction between herds that were open with respect to replacement animals, and herds that were closed in the 1st dimension. In regards to open herds, the 2nd dimension of both MCA solutions revealed general patterns in introduction and transportation practices, where one quadrant was associated with good biosecurity practices and the opposite quadrant was associated with poorer practices. The findings from this study emphasize that evaluating farms in terms of how they perform on any one biosecurity practice has some limitations. Investigating which practices tend to occur together to form strategies is much more informative, and provides further insight into a producer’s overall approach to biosecurity.

## Competing interests

The author declares that they have no competing interest.

## Authors’ contributions

KB and ZP conducted the statistical analyses and wrote the manuscript. CD reviewed the paper and provided comments regarding the discussion & conclusions. RD provided statistical support and reviewed the paper. DH provided information about the methodology for data collection and reviewed the paper. RF organized the survey, reviewed the paper, and provided comments regarding the discussion & conclusions. All authors read and approved the final manuscript.
